# Quantitative patterns of visual impairment and recovery in children with brain injury

**DOI:** 10.21203/rs.3.rs-4511323/v1

**Published:** 2024-07-30

**Authors:** Scott W.J. Mooney, Nazia M. Alam, Matthew J. Sciarabba, Kieran R. Sheldon, Glen T. Prusky

**Affiliations:** 1. Burke Neurological Institute, White Plains, NY, USA; 2. Blythedale Children’s Hospital, Valhalla, NY, USA; 3. Weill Cornell Medicine, New York, NY, USA

**Keywords:** Cortical visual impairment, pediatric neuroscience, brain injury, eye movements, contrast sensitivity, spatial vision, traumatic brain injury, brain injured children, measurement, psychophysics, visual field

## Abstract

Brain injury can cause many distinct types of visual impairment in children, but these deficits are difficult to quantify due to co-morbid deficits in communication and cognition. Clinicians must instead rely on low-resolution, subjective judgements of simple reactions to handheld stimuli, which limits treatment potential. We have developed an interactive assessment program called the Visual Ladder, which uses gaze-based responses to intuitive, game-like tasks to address the lack of broad-spectrum quantified data on the visual abilities of children with brain injury. Here, we present detailed metrics on eye movements, field asymmetries, contrast sensitivity, and other critical visual abilities measured longitudinally using the Ladder in hospitalized children with varying types and degrees of brain injury, many of whom were previously considered untestable. Our findings show which abilities are most likely to exhibit recovery and reveal how distinct patterns of task outcomes defined unique diagnostic clusters of visual impairment.

The impairment of visual function can negatively affect performance in many areas of life. Its impact is particularly salient in cases of brain injury, where deficits in ocular abilities, spatial vision, and perceptual interaction often co-occur with other neurological dysfunctions such as impaired cognition or loss of communicative ability^1,2^. These same co-morbidities make it difficult to assess precisely how – and how severely – visual function has been affected, as most tests rely on a minimum degree of both task comprehension and directed responses (usually verbal or mechanical) to infer visual abilities and thresholds. This is a core paradox in clinical neuroscience and a problem that is further exacerbated in children, who already find it more difficult to perform complicated or long visual tests than adults^3^. Information about the visual function of children with brain injury is therefore far more sparse than comparably injured adults.

In developed countries, the most common classification of visual impairment in children is cortical or cerebral visual impairment (CVI), which is estimated to affect 30–40% of children with visual disorders^4,5^ and more than 10% of children with impaired development^6,7^. As the name suggests, CVI is a monolithic category of disease with a large range of possible causes^8,9^ and comprises a vast, diverse range of possible specific deficits^10–13^ – most of which cannot be measured by any method other than qualitative observations of gaze reactions by trained assessors and carer/physician questionnaires^14,15^. A diagnosis of CVI typically also requires ocular and geniculate causes of impairment to be partially or completely ruled out^14^, and while lower-level ophthalmological symptoms (e.g., pupil dysfunction, strabismus, and nystagmus) are easier to assess in non-verbal children than complex, brain-based higher-level visual function deficits^16^, the co-morbidities of brain injury still pose a significant barrier to their diagnosis. Our understanding of any *subcategories* of CVI – let alone how to differentially diagnose, prognose, and treat them – is therefore constrained by a lack of quantitative data, despite the prevalence of this condition in children with brain injury.

We have previously described a vision assessment system called the Visual Ladder^17^. The Ladder comprises a sequence of interactive vision tests that use eye tracking to detect a subject’s responses to screen-based stimuli, dynamically adjust those stimuli in real time, and compute visual thresholds and other metrics based on the subject’s gaze and overall task performance. The tasks are “disguised” as fast, gaze-based video games to promote attention and encourage high-effort performance, which is particularly important when testing children (and testing them multiple times)^3^. Example screenshots from these tasks are shown in Figure 1. In our previous paper, we presented vision data from ten children with a variety of visual and neurological deficits and demonstrated the Ladder’s ability to quantify key visual metrics such as directional asymmetries in eye movements (saccades and smooth pursuits), directional visual field sensitivity, and the contrast sensitivity function (CSF)^17^. Though not every child could be tested with the Ladder (usually due to a weak eye tracker signal caused by frequently closed eyes, strabismus, or pathological nystagmus), we showed that many children who were previously considered “untestable” – including non-verbal children and children with a CVI diagnosis – could have key visual abilities precisely quantified through gaze responses to interactive, titrated stimuli^17^.

Here, we present a much larger dataset of vision scores collected and computed by the Visual Ladder in 98 children aged 3 to 19 from the Brain Injury Unit at Blythedale Children’s Hospital in Westchester County, New York. These children had a wide variety of neurological conditions, including arteriovenous malformation, traumatic brain injury, and perinatal hypoxia. Children were repeatedly tested with a regularity that depended on availability and flexibility in their hospital therapy schedule, school schedule, the child’s daily well-being, and their willingness to participate on that day. We also collected three Ladder sessions of normative data from ten children with no known visual or neurological conditions, who were tested at the Burke Neurological Institute in Westchester County, NY. We then performed an extensive analysis on this dataset to quantitatively (a) determine which vision scores are most likely to be impacted by brain injury, (b) detect clusters of children who generated similar patterns of task outcomes (and may therefore represent latent subcategories of visual impairment), (c) compare these scores and clusters with the Roman CVI Scale^8,18^, and (d) examine longitudinal changes and compare between-user and within-user variance on each score. Our aim was to enable pediatric neuroscience to quantitatively describe meaningful patterns of impaired visual function that may prove useful in the clinical diagnosis of children with brain injury, which promises to provide a base for more detailed prognosis and future condition-specific treatment.

## Results

The Visual Ladder software produces a large amount of data at multiple levels of computational depth, including various high-level metrics computed directly during specific tasks (visual field latency, contrast sensitivity, etc.), mid-level classified phenomena such as eye movement behaviours (saccades, pursuits, and fixations), and low-level per-eye gaze data captured at 90 Hz. We reduced this data to a practicable number of computed vision assessment scores chosen through a combination of prior knowledge and guidance from a principal component analysis (PCA) of specific data subsets. This process of score computation is described in detail in the Methods and ultimately produced 30 outcome scores, each computable from a single Ladder session.

### Score Histograms

Density histograms of scores for all children, averaged over all Visual Ladder sessions for each child, are depicted in Figure 2 for each of three groups: children at Blythedale who received a CVI Range score (red; max n = 23), children at Blythedale who did not qualify for a CVI Range score (green; max n = 75), and control children with no known visual or cortical impairment who each completed three sessions at Burke Neurological Institute (BNI) (grey; n = 10). Group means are marked by dotted lines and the name of each score is given above each plot. Recurring abbreviations “R:L”, “U:D”, and “H:V” represent bias scores computed between right vs. left, up vs. down, and horizontal vs. vertical directions for the indicated metric, respectively. Abbreviation “C:E” in the top-right plot corresponds to central vs. edge fixations, where central fixations were defined as fixations whose mean position was inside a centred box with dimensions that were half the display width and height (25% of total display area). The scores are sorted into two major sections: *general scores* (first four rows), which use varying score-dependent units, and *bias scores* (middle six rows) computed as the logarithm of binned directional score ratios (see Methods). This two-section layout is repeated for all multi-score analyses described below. Note that not all 98 children tested at Blythedale appear in every histogram, as some children were unable to engage with and/or generate enough data for the more difficult tasks after Bubble Burst (Moving Bubbles, Field Bubbles, and particularly Gradiate). The last row depicts the three external scores measured/provided for a subset of children at Blythedale: CVI Range centre score (the presence of which defines the “Blythedale CVI” group in all analyses), Physical Abilities and Mobility Scale (PAMS) score^19^, and Cognitive and Linguistic Scale (CALS) score^20,21^.

Many striking results are apparent in these histograms. One immediate observation is that the mean bias scores for children at Blythedale (red and green) do not tend to deviate from the control children (grey), likely because any direction-specific deviations from the normative asymmetries exhibited by different children tend to cancel out. The exception is Central vs. Edge Fixation Count (fifth row, first column), which is not a directional or cardinal asymmetry. Blythedale children were significantly more biased towards edge fixations than the control children (*t* = −2.72, *p* = 0.0076), and within Blythedale children, those with CVI scores (red) were significantly more biased towards edge fixations than non-CVI children (green; *t* = −4.71, *p* < .0001). This pattern of significant group differences repeats for almost all general scores (top section): Blythedale children usually deviated significantly from the control norms (black asterisks in the plots) and CVI children at Blythedale usually deviated more than non-CVI children (red asterisks in the plots). For the general scores, the valences of these deviations are clearly interpretable: Blythedale/CVI children exhibit *more impaired* outcomes on these scores than control/non-CVI children. These impairments include: less time fixating on the screen in Bubble Burst and more time fixating *off* the screen; less frequent and shorter saccades in Bubble Burst; less time pursuing in Moving Bubbles; greater mean latency and less mean directness when responding to targets in Field Bubbles; more eye tracker noise; more anisotropic pursuits; greater head tilt relative to the display; worse contrast sensitivity; and more time to make progress in Gradiate. Unsurprisingly, CVI children also had significantly worse mean PAMS and CALS scores than non-CVI children. The *t* and *p* values for these tests are given in Extended Data Table 1.

Among the eye movement bias scores, differences in *variance* between the three groups were surprisingly small to non-existent. In practice, there is likely an upper limit to some of these asymmetries for Ladder participants due to the zero-sum restriction of eye movement vectors in the limited visual space of the display: it is difficult, for example, to produce twice as many rightward as leftward saccades in Bubble Burst when saccades are the only way to return one’s gaze to the left side of the screen. In the case of pursuits measured during Moving Bubbles, however, this is not true: Ladder participants with asymmetric impairments may smoothly track every bubble moving rightward and fail to smoothly track those moving leftward, relying on saccades to navigate around the display as needed. This may explain the greater variance in pursuit bias scores (in all groups) compared to saccade bias scores. The similar variance *between* groups, however, is more surprising for the pursuit scores. It may be due to a combination of the shorter duration of Moving Bubbles and control children only completing three sessions of the Ladder. The ten targets presented during a single Moving Bubbles session may not be sufficient to reliably produce a normative bias score, and the means for children in the control group (calculated using only three such scores) may consequently exhibit more variance. The largest between-group differences in bias score variance occur in visual field latency, where children in the control group exhibit significantly less variance than children at Blythedale across all three directional comparisons.

To demonstrate how often and how severely scores from children at Blythedale deviate from the normative means attained by the control children, we transformed scores for the CVI and non-CVI children at Blythedale (except CVI centre, PAMS, and CALS, for which control children have no data) into “control Z-scores”. In Figure 3, all horizontal axes represent the number of standard deviations away from the control group’s mean for that score. A score of zero (central black dotted line) therefore represents a normative score. All horizontal axes range from Z-scores of −15 to +15, which allows the relative extent and direction of deviations from the control group to be compared across scores. The most severe impairments occur in mean visual field latency – where the mean score for all children at Blythedale is an extreme *ten* standard deviations above the normative latency – and Concuity/CSF contrast sensitivity, where the Blythedale means are 3 to 4 standard deviations below normative. These mean Z-score deviations are particularly large due to the consistency of the control group’s outcomes for these scores, e.g., a standard deviation of just 88 milliseconds for mean Field Latency. The relative degree of skew in each score is also apparent: there are a small number of children who exhibit extreme deviations in off-screen fixation time, horizontal vs. vertical field latency bias (particularly in the direction of horizontal impairment), Gradiate mean trial time, eye tracker noise, pursuit anisotropy, and head tilt.

### Impairment Patterns in Scores

The raw score histograms are highly informative, but *patterns* of outcomes exhibited by the children at Blythedale are more likely to inform specific clinical diagnoses. To investigate this, we analysed the Ladder scores using K-means clustering. This method divides participants into multiple clusters based on the similarity of their overall score configurations, which in the present study likely corresponds to interpretable kinds and/or grades of impairment. To ensure that the more impaired children were included in this analysis, we excluded the three scores computed from the Gradiate task, as only 67 children at Blythedale were able to perform this task at least once. Using the “elbow method”, we identified eight clusters as the appropriate cluster count (Extended Data Figure 3). This eight-cluster K-means analysis included 86 out of 98 children at Blythedale (excluding 12 who were missing a mean value for at least one score but including 17 out of 23 with CVI Range scores) and the ten control children.

Heatmaps of the score means for each cluster, coloured by their log absolute value in units of control group standard deviation (control Z-scores, as in Figure 3), are depicted in Figure 4. These clusters are readily interpretable and provide a comprehensive framework for evaluating each child’s Ladder performance through the lens of a practicable number of outcome groups. They are largely defined by the overall degree of deviation in the nine general scores (first three rows) and the Central vs. Edge Fixation Count score. The most relevant score is clearly Mean Field Latency from the Field Bubbles task, where non-impaired children exhibit such reliably fast means (see Figure 2) that children with minimal other deviations across the board are still split into two clusters based on a mean Field Latency increase from 0.46 seconds (cluster 1) to 0.6 seconds (cluster 2). The most impaired clusters exceeded mean Z-scores of 30 on this key score.

We refer to this dimension of overall general score deviation as *general vision impairment* (GVI). We have also graded it as “absent”, “mild”, “moderate”, or “severe” across the eight clusters – labels that correspond to mean cluster Field Latency values of approximately 0.5, 1, 2, and 3 seconds, respectively. In addition to differences in GVI, there are multiple clusters that we categorize as “mild GVI” that exhibit other specific, quantifiable deviations amongst the 18 bias scores. Cluster 4 (n = 5) exhibits *leftward neglect*, with large mean deviations in Right vs. Left Field Latency (z = −5.75) and Directness (z = 3.51). Cluster 5 (n = 5), however, exhibits *horizontal neglect*, with large mean deviations in Horizontal vs. Vertical Field Latency (z = 3.99) and Directness (z = −2.28), as well as Horizontal vs. Vertical Saccade Count (z = −2.45). Interestingly, this cluster also exhibits less direct – but not slower – responses to upward field targets than downward. Note that in terms of absolute bias, these deviations may not seem extreme – cluster 4 has a Right vs. Left Field Latency bias score of −0.72, indicating that they responded ~1.65 times slower to leftward targets than rightward, on average – but because children in the least impaired clusters performed so reliably symmetric, even this small absolute asymmetry was detected by our cluster analysis on Z-scores. Finally, cluster 6 contains just one child who appears to have a severe pathological nystagmus that produced an extreme Pursuit Anisotropy score (z = 8.02) and a wide array of asymmetric bias scores that are likely downstream of this. We independently confirmed that this child had a diagnosed pathological nystagmus.

To emphasize the combinations of scores that play the largest roles in defining these clusters, we selected six two-dimensional “slice” views of the eight clusters to depict in Figure 5. The clusters with minimal bias score deviations are coloured by GVI severity, from green (two shades: no detectable impairment or a mild Field Latency impairment only) to yellow (mild GVI) to orange (moderate GVI) to red (severe GVI). Other colours represent the three clusters that combine mild GVI with other specific impairment characteristics (middle row of Figure 4): leftward neglect (blue), horizontal neglect (purple), and the single child with a severe pathological nystagmus (grey). Child group is also indicated by marker type; the control group children (squares) are generally tightly clustered at the origin (the control group mean, by Z-score construction), while the children with CVI scores (crosses) are over-represented in the moderate and severe GVI clusters. The views provided by the first four graphs emphasize the spread of the mild, moderate, and severe GVI clusters, while the fifth and sixth graphs emphasize the defining characteristics of the mild GVI clusters with leftward (blue) and horizontal (purple) neglect, respectively.

Finally, while our cluster analysis excluded the three Gradiate scores to include the 29 children at Blythedale who could not complete the Gradiate task, we examined the mean Gradiate scores for those children who did have them in each cluster. Boxplots of the absolute mean Gradiate scores for the first seven clusters are shown in Extended Data Figure 4, along with the proportion of children in that cluster who could achieve Gradiate scores (left plot). All Gradiate scores tended to become more impaired and less reliably present in clusters with greater general vision impairment. None of the six children in cluster 8 (“Severe General Vision Impairment”) could complete the Gradiate task.

### Ladder Correlations with the CVI Range

We next sought to determine which if any of our 30 Ladder scores were correlated with the Roman CVI Range scores given to the 23 children who met the criteria for receiving this assessment at Blythedale. Can we predict a child’s CVI Range score based on their performance in the Visual Ladder, or does it form an independent dimension of assessment from the Ladder scores?

In line with the clusters identified above, and the over-representation of CVI-scored children in certain clusters, we tested correlations between the mean CVI Score centre (the value halfway between the low and high range outcomes awarded by our tester) and a subset of 16 Ladder scores: the nine non-CSF general scores, the six Field Latency/Directness bias scores, and the Central vs. Edge Fixation Count bias score. We confirmed that the CVI centre score is a valid simplified outcome of the Roman scale, as the upper and lower CVI Range scores awarded by our trained assessor were almost perfectly correlated (*r* = 0.997, *p* < .0001). We controlled the false discovery rate for these tests at α = 0.01 with the Benjamini-Hochberg procedure.

The CVI Range centre score was significantly correlated with three Ladder scores across the same 17 out of 23 CVI children as our cluster analysis, all related to fixations: Central vs. Edge Fixation Count bias (*r* = 0.775), Fixation Onscreen Time (*r* = 0.819), and Fixation Offscreen Time (*r* = −0.701), all unadjusted *p* < .001. A multiple linear regression of CVI Range centre score on these three scores further revealed that they together account for 68.8% of the variance in the CVI Range (*F* = 9.565, *p* = 0.001; Figure 6). This is substantial, considering that the Roman CVI Range score is computed from many different types of behavioural and report-based assessment, most of which have no direct relationship to fixation behaviour.

### Longitudinal Analysis and Recovery

The scope of this paper is predominantly focused on overall mean Ladder performance and the intrinsic group differences revealed by our cluster analysis. However, as knowledge of visual function recovery magnitude and likelihood – even in the absence of targeted vision therapy or treatment – is central to the usefulness of impairment classification, we performed an initial analysis of longitudinal change for all Ladder scores, all sessions, and all children at Blythedale using a mixed linear model approach (MLM). This model type allows random variables (here, child identity) to be combined with non-random variables of irregular frequency and spacing (time of Ladder session, in days since that child’s first session) as linear predictors of score value. We excluded Ladder sessions more than 290 days after that child’s first session, as only ten out of 98 children had sessions extending over more than this many days, with large gaps in testing due to being temporarily discharged from the hospital. To remove the influence of any early practice effects, we also excluded Ladder sessions that took place within 10 days of each child’s first session, which caused some children with short hospital stays to be removed from the analysis altogether. Data were again analysed as control group Z-scores; however, as deviations from normative scores for the 17 directional bias scores (i.e., all bias scores except Central vs. Edge Fixation Count) could occur in either direction, we used the absolute values of these bias Z-scores to test whether recovery occurred for children who may have initially presented with a bias in either direction.

The relationships between score value and relative session time are depicted in Figure 7. Every dot represents a single Ladder session for a child at Blythedale, coloured by their cluster membership from the preceding analysis. The horizontal axis represents relative session time, in days since the first session, and the vertical axis represents the score value in standard deviations from the control group mean. For each score, we performed separate two MLM analyses: one with the 29 Blythedale children who were members of clusters 1 or 2 in our preceding analysis (“no general vision impairment”; green) and one with all children from the other six clusters (“mild general vision impairment” or worse; red). The green and red dotted lines represent the linear relationship between score outcome and time for these two subgroups of children, respectively, and are only depicted as opaque for cases where this relationship was significant after controlling the false discovery rate at α = 0.01 with the Benjamini-Hochberg procedure across all 60 models. Coefficients of slope (*b*_30_, change in score per 30 days) and Y intercept (*y*_0_, score at relative time zero) are also depicted in these cases. The horizontal range for each model line covers the first 95% of all session samples by time for that group of clusters.

The models reveal that in almost every case where Ladder scores changed significantly over time, the direction of change was positive – i.e., *recovery* – with the score moving closer to the control group mean (z = 0). The only exception was mean Saccade Distance, where children in the more impaired clusters not only tended to present with shorter saccades than the control group, but made even shorter saccades over time; however, the slope of this change was only −0.058 control group SDs per 30 days. Notably, children whose *mean* scores across time placed them in our “no general vision impairment” or “mild field latency impairment only” clusters (both shades of green) also exhibited significant (though typically far lesser) recovery on some scores, indicating that many of these children did have a small amount of room to improve on the Visual Ladder at the start of their testing period.

The largest significant recovery deltas for the impaired clusters (red lines) occurred in mean Field Latency, Pursuit Anisotropy, and all three CSF scores, which remarkably recovered to near-normative levels. However, as discussed previously, many of the most impaired children could not engage with the Gradiate task well enough to produce these scores (though more children could generate CSF Concuity Sensitivity scores than CSF Contrast Sensitivity scores). Notably, the three scores associated most strongly with CVI Range Score – Fixation Onscreen Time, Fixation Offscreen Time, and Central vs. Edge Fixation Count – did not exhibit any significant change over time.

Finally, we aimed to compare between-subject variance of each score with within-subject variance across all children measured at Blythedale. The ratio between these two statistics provides an indication of how many estimates of each score (e.g., how many Ladder sessions) are needed to confidently place a given child in the population on that score: a score with high between-subject variance and low within-subject variance would need less samples than a score with low between-subject variance and high within-subject variance. We assessed this by analysing a mixed linear model for each score that included all children (not separated by cluster) and taking the model’s estimates of between-subject and within-subject residuals (as standard deviations). These residuals are depicted in Figure 8 as blue (between-subject) and red (within-subject). The background of each score’s plot is coloured based on the log ratio of the two residuals. A more blue background therefore indicates a score that can be established with comparatively more confidence with fewer samples. Overall, these residuals indicate that the general Ladder scores are easier to assess than the bias scores, where within-user variance tended to be much higher than between-user variance.

## Discussion

We identified, computed, and analysed 30 outcome scores from our Visual Ladder assessment program that provide a comprehensive picture of low-level visual abilities in children with varying types and degrees of brain injury. The primary outcome of our analysis is the identification of distinct evidence-based clusters that both (a) grade the severity of correlated deficits that we refer to as *general vision impairment* (absent, mild, moderate, or severe) and (b) flag the presence of additional, independent deficits such as a leftward vs. rightward bias in eye movements, a horizontal vs. vertical bias, or a pathological nystagmus (Figure 4).

These clusters are not just categorical labels – they are quantitatively defined and actionable. A child’s class of impairment, as graded by the Ladder, makes specific simultaneous predictions about their response time to peripheral visual stimuli, their sensitivity to low-contrast images, their ability to track moving stimuli in particular directions, their ability to fixate on “points of interest” placed in front of them (particularly at more severe grades of impairment), and more. These are behaviours that can be accounted for and potentially targeted for improvement by clinicians, all with consideration of their precise severity.

The distinctions between these Ladder grades are minor and unlikely to be measurable without the precision of eye tracking, automated real-time analysis, and immediate stimulus adaptation afforded by custom software. Mean response latency in Field Bubbles, for example, is so reliably low in normative children that even an increase of 100 ms was sufficient to define a separate grade of “nearly normative” performance in our analysis. Our findings indicate that impairment in this one score is the most useful, reliable, and universally attainable vision metric provided by the Ladder. The next most useful individual scores are likely the “fixation triad”: the ratio of central to edge-adjacent fixations and the relative amount of time spent making fixations both on and off the display. Our data reveal that making more frequent fixations away from the centre of the display, and even off the display, is a behaviour that is strongly associated with more severe general vision impairment. These three scores were also significantly predictive of a child’s score (when criteria were met^8,18^) on the Roman CVI Scale, accounting for 68.8% of variance. This unusual “inattentive” behaviour has clearly been noted by clinicians for a long time, but here we have precisely quantified it, allowing early classification of mild cases that may have previously eluded detection. Notably, none of these fixation scores demonstrated any mean recovery over time, which suggests that this more severe form of vision impairment may require specific intervention strategies over longer timelines than the 290-day limit of our analysis.

Scores of peak contrast sensitivity from Gradiate task – though only obtained at least once by 69% of children at Blythedale – remarkably exhibited significant and relatively steep recovery over time (Figure 7). We observed a similar degree of recovery in one case study we have published previously^17^, and it is encouraging to see this trend hold across a much larger dataset in a way that cannot be attributed solely to practice effects. For the impaired clusters, mean Gradiate trial time also significantly decreased back down to normative levels. We believe this is the first evidence of this phenomenon in a large population of children with brain injury, as contrast sensitivity has historically been one of the most difficult and time-consuming vision assessments to conduct accurately^22^. It suggests that spatial vision is one of the first visual functions to undergo recovery after a brain injury, and may require minimal intervention beyond the rich, broadband content presented by the world itself. The same recovery occurred for the one-threshold “Concuity” metric from Gradiate, which repeatedly assesses the same combined acuity/contrast sensitivity threshold using only a single ball. This simpler version of Gradiate allowed data collection from 73 children (85%) and therefore holds promise as a compromise between testability and in-depth CSF analysis in the future. It should also be noted that no subject ever obtained, in any session, a Gradiate CSF threshold beyond what would reasonably be considered plausible for a non-impaired human, which provides further evidence for the high resistance to false positives that we have previously noted about the Gradiate task^22^.

Finally, the sizes of the identified Ladder clusters also shed light on the relative prevalence of different grades and types of visual function impairment in children with brain injury. In fact, 29 of the 86 children at Blythedale (34%) who featured in our cluster analysis were graded as having no detectable general visual impairment or only a mild field latency impairment. A further 26 had mild impairment (30%), 11 had mild impairment combined with a directional bias or nystagmus (13%), 14 had moderate impairment (16%), and only 6 had severe impairment (7%). These relative cluster proportions may inform prior expectations for frequencies of visual impairment severity at other non-emergency pediatric sites.

The Visual Ladder has room for improvement, and much of the information collected and analysed in this study will be used to this end. First and foremost, we plan to automate and integrate much of the present analysis into the immediate outcomes of the Ladder, including classification into one of the graded clusters we have identified and the use of normalized Z-scores. Clinical users will then be able to use this more actionable, high-level output without needing to manually interpret the large volume of underlying eye tracking graphs and scores. However, a more detailed breakdown will still be available to identify and quantify rare but clear-cut cases of specific visual dysfunctions such as pathological nystagmus. We also plan to modify task durations and metrics to improve the reliability of several scores – particularly the bias scores, where within-user tended to outweigh between-user variation. Though they do not share the same impact as the more general visual function scores like mean field latency and contrast sensitivity, the emergence of smaller clusters distinguished by the presence of specific directional asymmetries reinforces the need for these visual function metrics. Finally, we plan to expand our analysis to consider more complicated gaze interactions, such as the real-time accuracy of smooth pursuit tracking movements. Such metrics are much more sensitive to calibration error – a persistent limitation in gaze-based tests – and require deeper design considerations than the scope of our present analysis allowed. With our initial Ladder analysis completed, future work will also have room to consider individual subject histories and examine the relationship between Ladder-graded outcomes, other medical data such as scans and injury types, and subject outcomes. The available data from PAMS and CALS reported here (Figure 2) suggest that this is a promising avenue for dataset expansion.

Our data on visual function recovery, in tandem with anecdotal reports of improvement from several parents at Blythedale, raise the possibility that the Visual Ladder task could itself be therapeutic. This is clearly something that we cannot confirm without a controlled experiment, although this is complicated somewhat by the need for a Ladder-like approach itself to *measure* any improvements in vision that it may be causing. One future possibility is a study in which some children are tested with the Ladder more frequently than others, with the hypothesis that more frequent tested induces greater recovery over the same period of time – but the irregular and unpredictable nature of each child’s hospital schedule and length of stay makes this difficult. Expansion to multiple testing sites would likely make this hypothesis easier to test.

The visual system is our cardinal sense. If visual function is relatively intact, it can serve as a portal for delivering tailored therapeutic interventions and experiences. For a child with poor visual function, that opportunity is lessened, but the ramifications of this across both basic and clinical pediatric neuroscience will not be evident without the ability to assess visual function accurately. The Visual Ladder does not measure all dimensions of visual function, but it provides a quantitative foundation of assessment in many of the visual abilities that underpin those dimensions: oculomotor behaviour, attentional fixations, saccade response time, and contrast sensitivity. Many of the children in our study had specific numbers put to these abilities for the first time in their lives post-injury – and in some cases, those numbers revealed surprisingly intact visual function. Though not investigated here, it is likely that adults can benefit from the rapid, detailed information provided by the Ladder as well, particularly when their impairments preclude the use of traditional tests. We anticipate that the Visual Ladder and our present findings will aid ongoing efforts to classify, quantify, and eventually treat visual impairment across wide populations.

## Methods

### Subjects

**Extended Data Figure 1.**
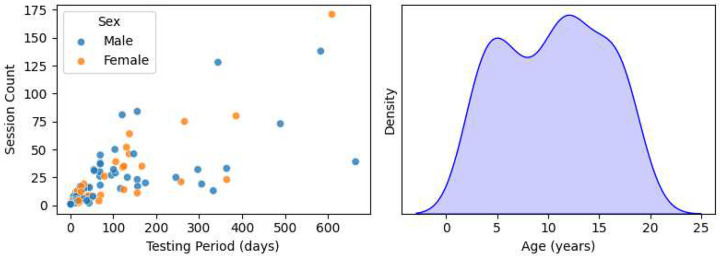
Scatter plot of session count vs. testing period in days, coloured by gender (left) and a density histogram of age distribution (right) for all 98 children at Blythedale who participated in the study.

108 children between the ages of 3 and 19 years were recruited between January 2021 and October 2023 through doctor or staff referral from the in-patient population at Blythedale Children’s Hospital in Westchester County, NY., USA. The eligibility criteria were a diagnosis of brain injury, the ability of the child to keep their eyes open, and the ability of the eye tracker to reliably detect their gaze. Ten children who ultimately could not satisfy this final criterion (i.e., could not keep their eyes open and/or exhibited too severe a strabismus for the eye tracker to handle) were excluded from the study after the initial testing attempt. The remaining 98 children (39 female) had a mean age of 10.47 years and SD of 4.96 years. The number of testing sessions and total testing period for each child depended on their length of hospital stay, their availability, and their condition. We aimed to test each child roughly once per week, but actual testing frequency was largely determined by individual patient schedules. Plots of the distributions of subject ages, genders, testing period, and total Ladder session count are depicted in Extended Data Figure 1. Note that Blythedale accepts patients below the age of 21; although the subjects aged 18–19 were not legally “children”, we refer to all subjects as children for simplicity. Ten children (9 female) with no known visual or cortical impairment (mean age 12.20 years, SD 7.27) were tested as controls in three sessions over the course of one month at the Burke Neurological Institute in Westchester County, NY, USA. These children were recruited from colleagues and family at the Institute.

### Apparatus

A widescreen 27-inch LCD Dell Optiplex 7760 all-in-one computer running Windows 10 was attached to a mobile trolley with a customized articulated arm. A portable battery was used to power the computer while it was moved around the hospital before being connected to an AC outlet for each test. A Tobii 4C eye tracker (50–95 cm operating distance; 90 Hz sampling rate) with a professional-level license (Tobii Technology, Stockholm, Sweden) was mounted at the base of the display and controlled with the Tobii Pro SDK library, which allows access to and recording of the display gaze point for each individual eye and the coordinates of each eye in world space. Raw gaze point data were smoothed with a denoising algorithm that examines frame deltas to avoid smoothing over true saccades. An estimate of mean valid gaze was then computed on each frame by taking the average of whichever eyes were valid on that frame. Stimulus behaviour was controlled in Python using the Shady graphics toolbox^23^, which was also used to calibrate screen gamma. Audio feedback was controlled with the Audiomath toolbox^24^. Minimum and maximum screen luminance values of 10.0 cd/m^2^ and 221.1 cd/m^2^ were measured, respectively, under controlled room illumination with an ILT1700 radiometer (International Light Technologies, Peabody, MA). Subjects were assessed at a distance of approximately 620 mm as possible. No other form of distance enforcement or head restraint was used, as this was impractical with children in a hospital setting. However, the software automatically blanked the screen, suspected data acquisition, and displayed a corrective message whenever the user’s eyes were closer than 520 mm or further than 720 mm from the display. At 620 mm, the Optiplex display subtended horizontal and vertical visual angles of 51.5 and 30.4 degrees of visual arc, respectively.

### Stimuli

The Visual Ladder program was constructed from four unique gaze-based tasks designed to elicit and assess different ocular and visual abilities:

- **Bubble Burst**: up to five simultaneous coloured bubbles move around the screen and pop when fixated upon, which prompts the user to generate a multitude of saccades for assessment.
- **Moving Bubbles**: large single bubbles appear and move randomly along a large number of possible preset paths and must be smoothly tracked to pop, which prompts the user to generate smooth pursuits in varied directions for assessment.
- **Field Bubbles**: small bubbles appear one at a time in random order at predetermined peripheral locations; the latency and directness of response saccades are used to assess visual field sensitivity.
- **Gradiate**: noise patches of varying contrast and spatial frequency content drift around the screen, and the user’s ability to see each patch is inferred from smooth gaze tracking (19).

**Figure 1.**
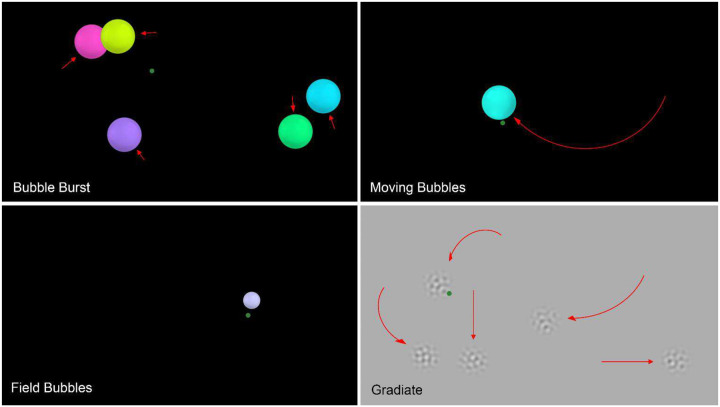
Example screenshots from the four main tasks of the Visual Ladder. In Bubble Burst (top-left), up to five coloured bubbles appear simultaneously and drift randomly across the screen. The subject must fixate these bubbles to pop them, encouraging isotropic fixations and saccades. In Moving Bubbles (top-right), bubbles appear one at a time and must be tracked with smooth pursuits to pop. In Field Bubbles (bottom-left), small static bubbles appear one at a time to assess directional saccade response latency and accuracy. In Gradiate (bottom-right), either one or five changing noise targets drift across the screen and become progressively harder to see (by varying spatial frequency and contrast) as they are tracked with smooth pursuits, ultimately generating specific CSF thresholds. The green marker in each screenshot represents an example gaze point (not shown during the task) and the red arrows indicate smooth stimulus motion.

**Figure 2.**
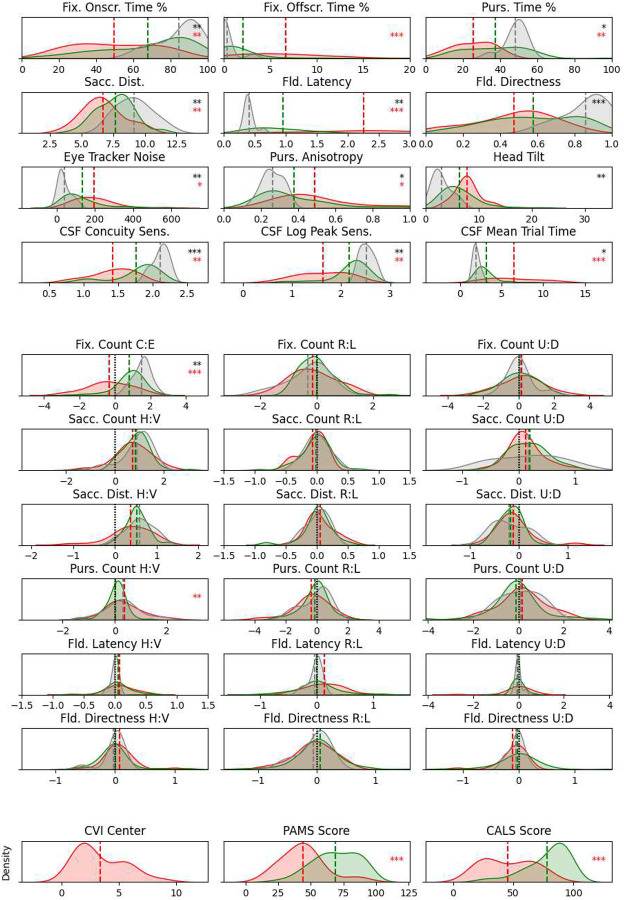
Score histograms for Blythedale children with CVI Range score (red), without CVI (green), and control children (grey). Group means are marked with dotted lines. Abbreviated score names are given above each plot. The full names of scores can be seen in the first column of **Table 1**, as well as the units of each score (horizontal axis). The numbers of CVI and non-CVI children who generated a mean for each score are given above the corresponding plots in Figure 3. The number of children in the control group is always ten for the 30 Ladder scores. To aid interpretation, the twelve “general scores” (first four rows) are visually separated from the 18 bias scores (middle six rows). This two-section layout of scores is consistent in all similar arrays of plots that follow. The last row shows the distributions for three non-Ladder scores measured only at Blythedale: CVI Range centre score (left), PAMS score (middle), and CALS score (right). Black asterisks indicate a significant difference between the control group and all Blythedale children combined (red and green) and red asterisks indicate a significant difference between CVI and non-CVI children at Blythedale; * p < .05, ** p < .01, *** p < .001.

**Figure 3.**
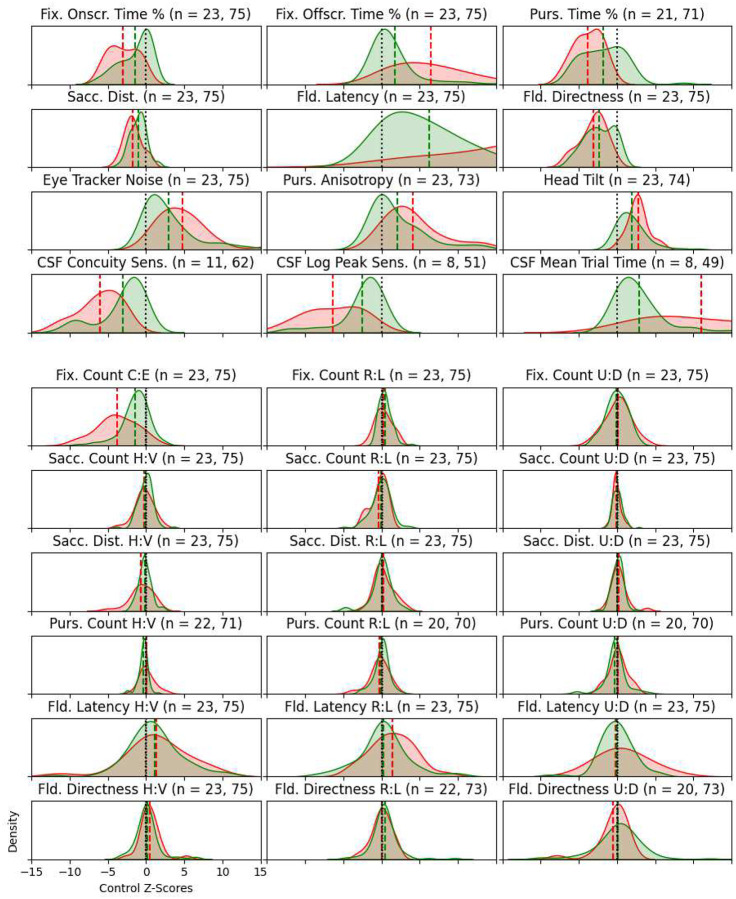
Density histograms of scores from CVI (red) and non-CVI (green) children at Blythedale, transformed into control group Z-scores. All horizontal axes are scaled to show the same range of Z-scores (−15 to +15) so that the relative extent and direction of deviations from the various normative scores is apparent. The numbers of CVI and non-CVI children that appear in each histogram are given above each plot. Note that the mean Field Latency z-score for the CVI group was 21.0, which lies beyond the right edge of the axis limits for that measure.

**Figure 4.**
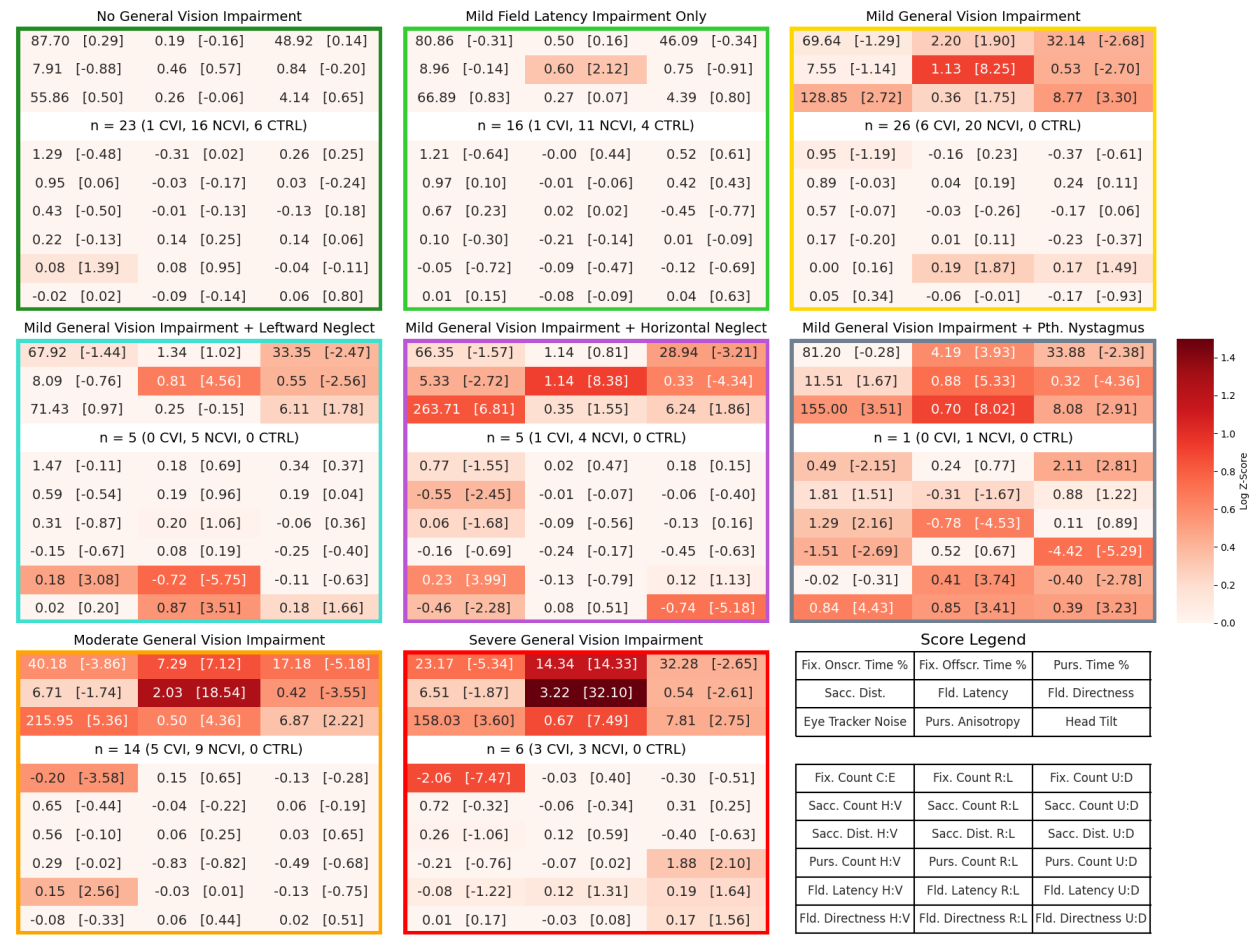
Heatmaps of mean scores for eight K-means clusters containing the 96 children with values for all 27 non-Gradiate scores. Clusters are labelled based on the patterns of deviations they manifest and are sorted manually from least to most “general vision impairment” in the first three rows of scores. The label in the middle of each heatmap also provides the total cluster size and a breakdown of that cluster’s size into CVI, non-CVI, and control children. Cells represent scores in the layout indicated in the bottom-right plot. Each cell indicates both the raw value of its mean, in units of that score (e.g., percentage of time in the first row), and the control Z-score in square brackets. To depict the deviations of cluster means properly and consistently across all cluster – some of which contain extreme values – cells are coloured from white to dark red by their log absolute Z-score.

**Figure 5.**
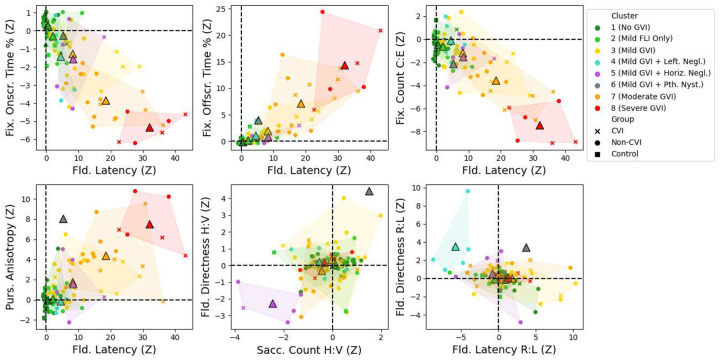
Six selected 2D slice views of selected Ladder score pairs (as control group Z-scores), with dot colours and corresponding convex hulls representing the six clusters produced by our eight-cluster K-means analysis. Marker type represents the group of each child: Blythedale CVI (cross), Blythedale Non-CVI (circle), or Control (square). Cluster centroids are marked with triangles. Note that the grey triangle represents the single child in the sixth cluster, who has a pathological nystagmus.

**Figure 6.**
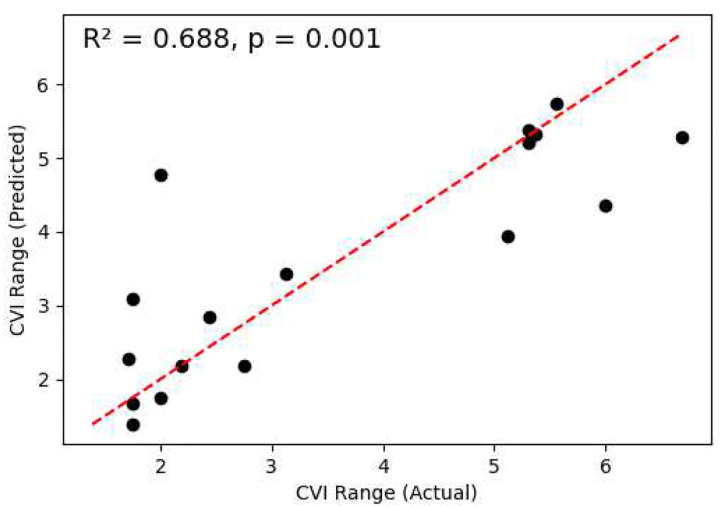
CVI Range centre scores assessed directly (horizontal axis) and the score predicted via multiple linear regression from three Ladder scores: Centre vs. Edge Fixation Count bias, Onscreen Fixation Time, and Offscreen Fixation Time (vertical axis; n = 17). The line of best fit is depicted in red and the model fit statistics are given in the top-left corner.

**Figure 7.**
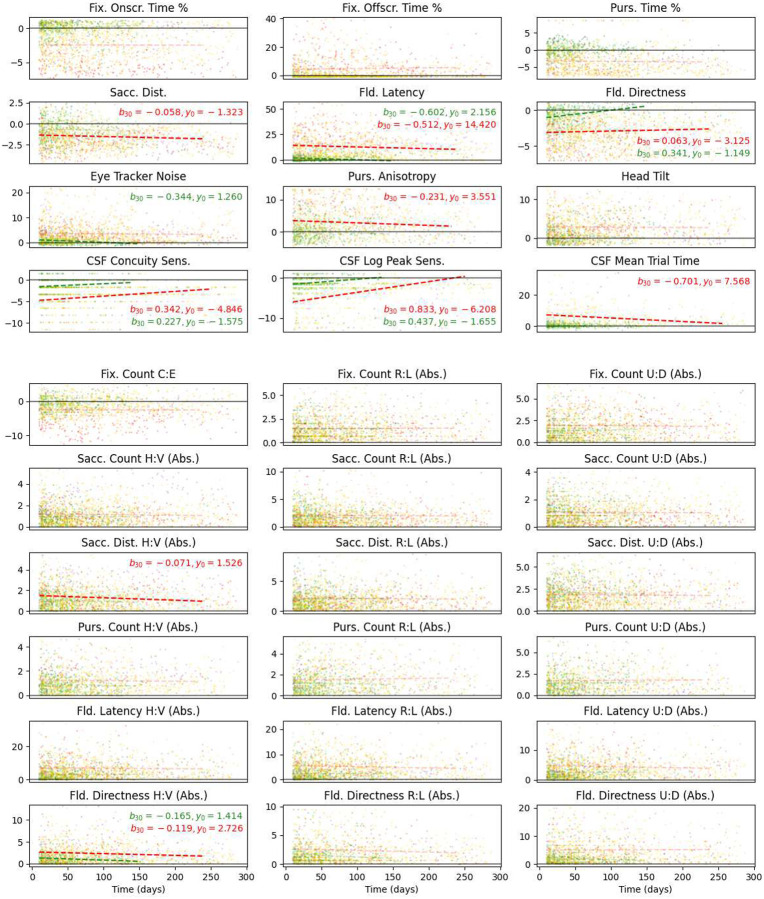
Scatter plots and mixed linear models of all children tested at Blythedale on all Ladder scores, arranged in the same two-section layout as previous figures. Every dot represents a single Ladder session for a single child, with child ID serving as a random variable in the models. Data are coloured by cluster as per Figure 4. The horizontal axis represents the relative time of the Ladder session in days after that child’s first Ladder session, which served as a non-random variable in the models, and the vertical axis represents score values as control group Z-scores. Note that the 17 directional bias scores were analysed as absolute values. Analysed session time ranged from 10 days (to discount early practice effects) to 290 days. The dotted lines represent the best-fit relationship between time and score outcome for the clusters 1 and 2 combined (“no GVI”; green) and all remaining clusters combined (“mild to severe GVI”; red). These lines only cover the first 95% of session data points and are only opaque for scores where this relationship was significant at *p* < .005. In these cases, the coefficients of slope (*b*_30_, the change in score per 30 days) and intercept (*y*_0_, the mean first Ladder score) are also given. The control group mean (Z-score zero) is marked with a black horizontal line on each plot.

**Figure 8.**
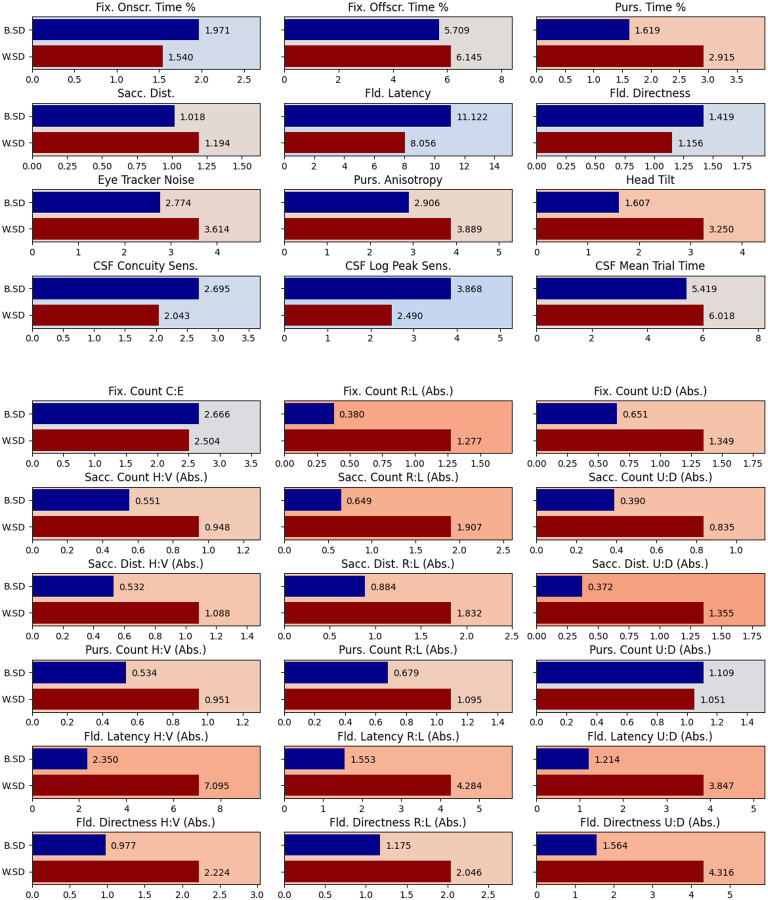
Between-subject residual standard deviation (blue) and within-subject standard deviation (red) for the mixed linear models constructed for each Ladder score from all Blythedale children, after accounting for time since first Ladder session as a non-random variable. Note that these models were not split by cluster as they were in Figure 7. The background of each plot is coloured based on the log ratio of the two metrics: a more blue background indicates relative more between-subject variance, while a more red background indicates relatively more within-subject variance.

Two variants of the Gradiate task were included in the program: (a) a variant that used one large, single target to measure CSF thresholds along the same sequence five times – a diagonal CSF vector we call **Concuity**, which lies approximately halfway between isolated measurements of acuity (horizontal vectors) and contrast sensitivity (vertical vectors) – and (b) a full five-threshold variant that sampled the full CSF with five simultaneous targets per trial. Screenshots of the tasks are depicted in Figure 1.

These five tasks ran automatically in sequence (following a brief practice task featuring four static bubble targets to pop) after the program was launched by the experimenter. An easier full-screen version of Gradiate was also run between Field Bubbles and Gradiate (see Mooney et al., 2021^17^), but as we have not yet addressed the higher false positive rate of this variant to our satisfaction (caused by the lack of any positional requirement for tracking detection), it was not included in the present analysis or discussion. The tasks were designed to appear as games: they combined real-time visual stimulus manipulation, eye tracker input (including denoising procedures and eye movement classifiers), and engaging audio feedback in the form of music and interactive sound effects. A random music track from a collection of songs was played in the background of each task. The goal was to design positive testing experiences that could plausibly be repeated many times for children, rather than a less interactive experience that could fail to motivate participation after the first session. As another key to sustained motivation in children is brevity^3^, our Ladder tasks were designed to adapt to detected eye movements as quickly as possible. Task time limits and trial limits were refined through pilot testing to avoid fatigue while collecting as much useful data as possible. A more detailed description of each task is available in Mooney et al. (2021)^17^ and, for Gradiate, in Mooney et al. (2020)^22^.

### Procedure

Subjects were tested at Blythedale Children’s Hospital in their rooms, either sitting up in wheelchairs or lying in bed according to their needs. The display was positioned approximately 620 mm in front of the child, perpendicular to their line of sight, using the mobile trolley’s articulated arm. Subjects were asked to remain as still as possible during the procedure to maintain this distance. The experimenter made attempts to account for unexpected head or body movements by making minor adjustments to the display position or angle. Session time of day varied, both within and between children, due to their often-unpredictable schedules. As subjects were often tested surrounded by other equipment and other children, room illumination was not controlled or measured; however, direct sunlight was avoided, and curtains were drawn when possible. We have previously found that variation in artificial room illumination does not significantly impact the results of our contrast sensitivity assessments^25^. Children were always awake and fed prior to testing and were thus adapted to photopic conditions.

Each Visual Ladder session began with a simple calibration step and practice bubble task to confirm the eye tracker was detecting gaze and allow the subject to “warm up”. The calibration procedure displayed a white gear subtending 5° in the centre of a black background on the display. Mean gaze was monitored and used to update an internal calibration offset when it was within 5° of the gear’s centre, which also caused the wheel to gain rotational speed. After two seconds of calibration, the fear disappeared. This calibration step sometimes took up to several minutes, and gaze was likely miscalibrated in numerous sessions (particularly for the more impaired children), but our tasks were designed to be resistant to even moderately large calibration errors (e.g., by relying more on first derivative gaze information).

The Ladder proceeded uninterrupted in most testing sessions, but the tester had a wireless keyboard with several controls available to respond to any unpredictable situations that often arose in testing:

- They could toggle a trio of small dots representing left, right, and mean gaze point on the display to debug cases of missing gaze (e.g., due to unusual difficulty in determining screen position). These gaze markers were used temporarily to check the status of the eye tracker, typically during the practice task, and were turned off when the tester was satisfied with the apparatus setup.
- They could toggle a readout of live user distance to check if the subject was at approximately 620 mm distance. This was enabled as needed if the child could not remain still for the session and required adjustment.
- They could end a task prematurely and proceed to the next task. Each subject’s available testing time was sometimes as short as ten minutes; the Ladder can be completed in that time by a child without severe impairment, but tasks can take much longer if every trial is left to time out (usually due to extended inattention). The option to skip tasks was added to ensure that impaired children with shorter time slots were able to try later tasks, such as Field Bubbles and Gradiate, without having to ensure long sequence of timed-out failures in Bubble Burst or Moving Bubbles.
- They could toggle the music at any time. Music seemed to improve task engagement in most cases, but for some (more severely impaired) children, was often a source of distraction.

The experimenter freely encouraged subjects to engage with the procedure and praised their performance, regardless of whether the child was verbal, and interacted freely with communicative children. Rarely, a session was ended early due to the subject’s time constraints or a health issue that prevented further participation. No cases of severe adverse health effects were attributed to any part of the experimental procedure. Experimental data were secured and managed with the REDCap database^26^.

### Score Selection and Construction

Some Ladder outcomes and statistics are of distinct interest, as they are known to be highly relevant to visual impairment. These include CSF shape metrics from Gradiate (specifically, log acuity and log peak sensitivity), mean values and directional asymmetries in visual field latency and directness from Field Bubbles, and the proportion of time actually spent performing the ocular behaviours required to make progress in Ladder tasks (e.g., on-screen fixation time, off-screen fixation time, and saccade rate in Bubble Burst; pursuit time in Moving Bubbles). However, after determining that CSF log acuity and log peak sensitivity were highly correlated – which is not surprising in a population for whom acuity-specific impairment (e.g., refractive error) is likely only a small source of variation – we chose to use only CSF log peak sensitivity in our analyses below.

We then sought additional scores that accounted for the largest amount of variance in mid-level ocular behaviours in children at Blythedale. *A priori*, we expected the most useful statistics in this domain to capture broad directional asymmetries (horizontal vs. vertical, right vs. left, and up vs. down) in the frequency and properties of the three types of ocular event automatically detected by our software: fixations, saccades, and smooth pursuits.

We binned saccades by their relative direction into four 90-degree-wide bins – right, up, left, and down – and computed *bias scores* for the count, mean distance (amplitude), and mean peak velocity of all saccades in each bin, as well as combined horizontal (right + left) and vertical (up + down) bins. Each bias score *S* was calculated as $S={\text{log}}_{2}(\frac{a}{b})$ for each pair of binned statistics *a* and *b*. If twice as many rightward saccades occurred as leftward, for example, the Right vs. Left Saccade Count score for that session would be *log*_2_(2) = 1. Conversely, if twice as many leftward saccades occurred, the bias score would be *log*_2_(0.5) = −1. We binned and calculated bias scores for smooth pursuits made during Moving Bubbles in a similar way, but because recorded smooth pursuit events varied immensely in duration and include many different directions in a single event, we first pre-processed these recorded movements by partitioning each event into *smooth pursuit segments* with a maximum duration of 0.25 seconds. This ensured that the actual time spent making pursuits in different directions by each child – even during the same continuously recorded ocular pursuit motion – was more accurately reflected in the binned statistics. We binned and computed bias scores for fixations based simply on their absolute mean position on the screen: right vs. left of centre and up vs. down of centre. However, instead of a horizontal vs. vertical bias score for fixations (which is ill-defined), we computed a central vs. edge bias score, where “central” fixations were defined as fixations that occurred in the central 25% area of the screen (i.e., a centred box measuring half display width by half display height). Finally, we binned and computed bias scores for both the latency (in milliseconds) and directness (proportion of trials in which the user directly saccaded to the target) measures from Field Bubbles into the same four 90-degree directional bins, including the horizontal vs. vertical combined comparison.

**Extended Data Figure 2.**
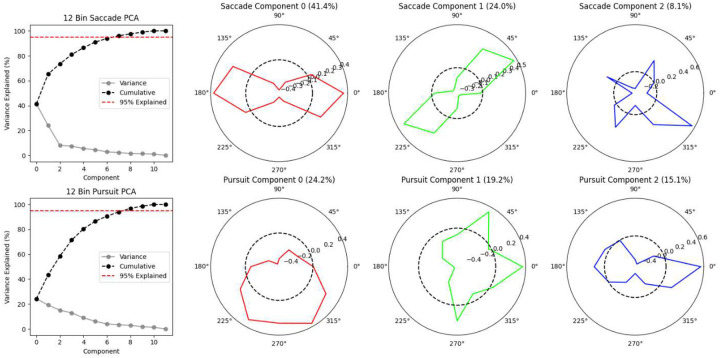
Principal component analyses of the relative proportion of eye movements in 12 binned directions for saccades during Bubble Burst (top row) and pursuits during Moving Bubbles (bottom row). The first column depicts the scree plots for the PCAs and the remaining three columns depict the binned directional loadings (i.e., eigenvectors) of the first three components for each eye movement. The dotted circle in each polar loading plot represents a neutral loading of zero. These loadings demonstrate the primary importance of opposing directional asymmetries in accounting for our data.

To confirm that relative differences in events across these broad directional bins were indeed responsible for a large proportion of the overall variance in ocular events, we performed a PCA on a higher-resolution directional dataset created by binning the relative directional frequency of saccades and pursuits into twelve bins, rather than four, and examined the loadings of the PCA components on these bins (Extended Data Figure 2). These loadings confirmed our expectations: horizontal vs. vertical cardinal asymmetry drives a dominant first component for saccades (41.4% variance explained) and a similar but oblique mirrored asymmetry drives the second component (24.0% variance explained). The first three components for pursuits are also clearly cardinally oriented, appearing to represent asymmetrical biases in favour of downward pursuits (24.2% variance), against leftward pursuits (19.2% variance), and simultaneously in favour of rightward pursuits and against vertical pursuits (15.1% variance), respectively. The bulk of between-user variance captured by these dominant PCA components is likely to also be captured by our four-bin directional bias scores.

We also computed the following pertinent scores:

- Saccade rate (saccades per minute): individuals with traumatic brain injury have been shown to saccade less frequently during eye tracking tests^27,28^ and real-world tasks such as walking^29^.
- Mean duration of successful Gradiate trials: a potential indirect measure of on-task attention and directed smooth pursuit ability (conditional upon the ability to complete the task at all).
- Pursuit anisotropy (magnitude of mean Cartesian pursuit direction): subjects with a pathological nystagmus or higher-level directional impairment are less likely to exhibit isotropic smooth pursuit events (i.e., with similar frequency across all spatial directions).
- Head tilt (mean absolute head rotation around viewing axis): subjects with symptoms of lateral impairments, such as asymmetric muscle control or ocular tilt reaction^30,31^, may be more likely to chronically tilt their head relative to the display, despite the best efforts of the tester to rotate the screen to match the user’s head orientation.
- Eye tracker noise: children who struggle to keep their eyes open, have ocular impairments such as strabismus or amblyopia, or otherwise exhibit attentional deficits that cause them to exhibit less interest in facing the screen are more likely to produce noisy eye tracker signals. We used the rate of “false positive saccades” as a measure of eye tracker noise: spatial discontinuities in gaze that were determined from context to not be saccades, e.g., as they were not preceded or followed by a fixation event.

### Statistics

Binned directional eye movements were analysed using principal component analysis (PCA) as described above. Subject group means on each computed Visual Ladder score were compared with two-tailed independent t-tests. Clusters of subjects were identified from Ladder score means using K-means clustering analysis (K = 8). Correlations between Ladder scores and Roman CVI Scale scores were analysed with Pearson correlation, with the false discovery rate controlled at 0.01 with the Benjamini-Hochberg procedure. CVI Scale score was regressed upon three Ladder fixation scores with multiple linear regression. Recovery models for Ladder scores were analysed using mixed linear models, with subject identity as a random variable and relative session time as a non-random variable. The false discovery rate for these models’ significance was controlled using the Benjamini-Hochberg procedure.

### Study approval

Parents/legal guardians of each child gave signed informed consent under an approved Institutional Review Board protocol managed by the Biomedical Research Alliance of New York (BRANY). All communicative children (as determined by their doctor) gave verbal assent, signed assent, or signed consent, depending on their age and ability prior to being enrolled in the study.

## Extended Data

**Extended Data Figure 3.**
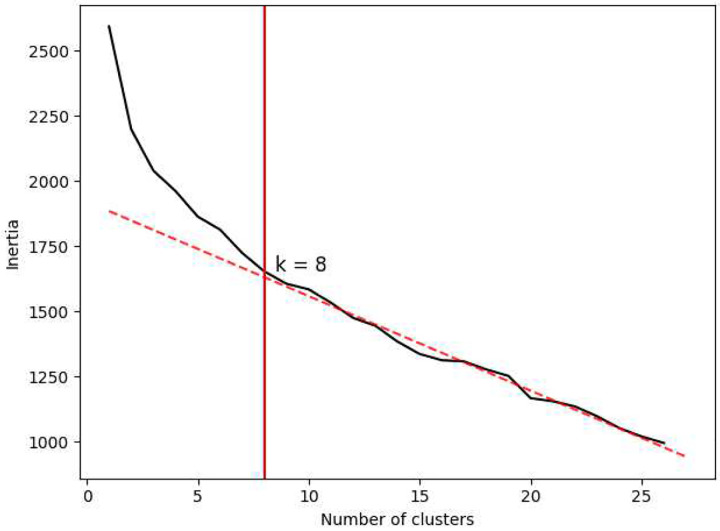
Inertia as a function of K-means cluster count (ranging from 1 to 27) for the 27-dimensional dataset with no Gradiate scores. This graph identifies k = 8 clusters as the optimal count, which is marked with a vertical red line. The dotted red line highlights how this cluster count marks the “elbow” point where inertia fall-off transitions to a linear slope.

**Extended Data Figure 4.**
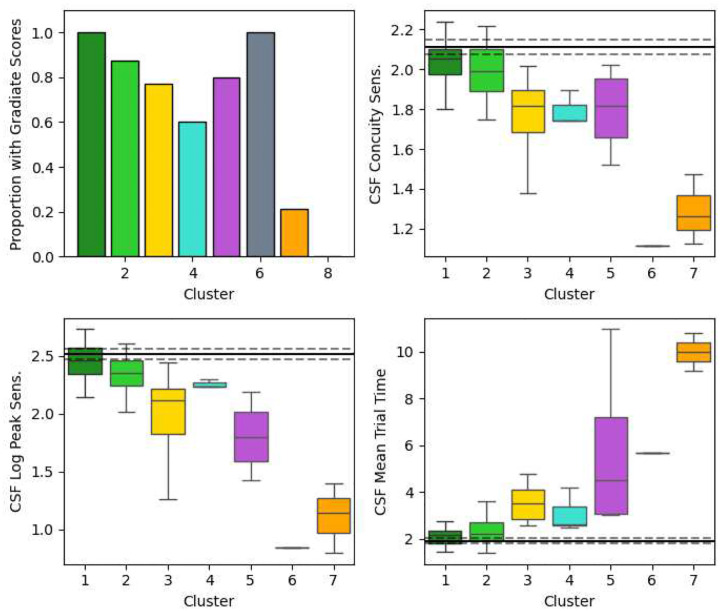
The proportion of children in each cluster who generated the three Gradiate scores (top-left) and boxplots showing the distributions of those three scores within each cluster. The solid and dotted horizontal black lines represent the mean and SEM of the control group on that score. Note that no child in cluster 8 could generate Gradiate scores, and that while the single child in cluster 6 (grey) did engage with Gradiate in at least one session, their pathological nystagmus would have severely impacted their outcomes.

**Extended Data Table 1.** Names, units, and statistics for t-tests on between-group mean differences for all 30 Ladder scores in addition to PAMS and CALS scores. One, two, or three asterisks indicate a significant difference at *p* < .05, .01, and .001, respectively.

Score	Units	Injury vs. Control		CVI vs. Non-CVI	
		*t*	*P*	*t*	*P*
Fixation Onscreen Time %	Percentage of time	−2.64	0.0094**	−3.25	0.0016**
Fixation Offscreen Time %	Percentage of time	1.88	0.0623	4.53	<0.0001***
Pursuit Time %	Percentage of time	−2.49	0.0146*	−3.07	0.0028**
Saccade Distance	DVA	−3.30	0.0013**	−2.63	0.0099**
Field Latency	Seconds	3.01	0.0033**	7.67	<0.0001***
Field Directness	Proportion [0, 1]	−4.10	0.0001***	−1.83	0.0699
Eye Tracker Noise	Jumps per second	3.01	0.0033**	2.31	0.0230*
Pursuits Anisotropy	Magnitude [0, 1]	2.14	0.0351*	2.29	0.0244*
Head Tilt	Degrees	3.34	0.0012**	1.71	0.0901
Concuity Sensitivity	Log10 sensitivity	−3.46	0.0009***	−3.01	0.0036**
CSF Log Peak Sensitivity	Log10 sensitivity	−2.81	0.0064**	−3.29	0.0017**
CSF Mean Trial Time	Seconds	2.48	0.0157*	4.84	<0.0001***
Fixation Count C:E	Log2 bias	−2.72	0.0076**	−4.71	<0.0001***
Fixation Count R:L	Log2 bias	1.30	0.1962	−0.87	0.3865
Fixation Count U:D	Log2 bias	−0.02	0.9850	0.53	0.6007
Saccade Count H:V	Log2 bias	−0.43	0.6715	−0.71	0.4777
Saccade Count R:L	Log2 bias	−0.38	0.7015	−0.90	0.3709
Saccade Count U:D	Log2 bias	0.10	0.9232	−0.80	0.4230
Saccade Distance H:V	Log2 bias	−0.86	0.3904	−1.87	0.0652
Saccade Distance R:L	Log2 bias	0.13	0.9007	0.78	0.4394
Saccade Distance U:D	Log2 bias	0.34	0.7357	0.94	0.3489
Pursuit Count H:V	Log2 bias	−1.10	0.2722	2.75	0.0072**
Pursuit Count R:L	Log2 bias	−0.26	0.7947	−1.05	0.2982
Pursuit Count U:D	Log2 bias	−0.37	0.7096	0.98	0.3308
Field Latency H:V	Log2 bias	0.95	0.3452	0.29	0.7701
Field Latency R:L	Log2 bias	0.50	0.6156	1.52	0.1309
Field Latency U:D	Log2 bias	−0.11	0.9128	−0.06	0.9516
Field Directness H:V	Log2 bias	0.42	0.6740	1.02	0.3107
Field Directness R:L	Log2 bias	0.56	0.5755	−0.99	0.3259
Field Directness U:D	Log2 bias	0.02	0.9809	−0.89	0.3753
PAMS Assessment	External score	N/A	N/A	−4.40	0.0001***
CALS Assessment	External score	N/A	N/A	−5.34	<0.0001***

## Data Availability

Mean values for the 30 computed Ladder scores for each subject are available via an Open Science Foundation repository at: https://osf.io/mzykt/?view_only=8e2459a2abe54967b2026c7978fcb064. The anonymized high-volume eye movement data used to compute these scores, including classified fixation, saccade, and pursuit events, can be made available upon request to the authors.

## References

[1] Good W. V., Jan J. E., DeSa L., Barkovich A. J. & Groenveld M. Cortical visual impairment in children. Survey of Ophthalmology 38, 351–364 (1994). 8160108 10.1016/0039-6257(94)90073-6

[2] Huo R., Burden S. K., Hoyt C. S. & Good W. V. Chronic cortical visual impairment in children: aetiology, prognosis, and associated neurological deficits. British Journal of Ophthalmology 83, 670–675 (1999). 10340973 10.1136/bjo.83.6.670 PMC1723072

[3] Witton C., Talcott J. B. & Henning G. B. Psychophysical measurements in children: challenges, pitfalls, and considerations. PeerJ 5, e3231 (2017). 28507816 10.7717/peerj.3231 PMC5429739

[4] Hatton D. D., Schwietz E., Boyer B. & Rychwalski P. Babies Count: The national registry for children with visual impairments, birth to 3 years. Journal of American Association for Pediatric Ophthalmology and Strabismus 11, 351–355 (2007). 17689825 10.1016/j.jaapos.2007.01.107

[5] Pehere N., Chougule P. & Dutton G. N. Cerebral visual impairment in children: Causes and associated ophthalmological problems. Indian J Ophthalmol 66, 812–815 (2018). 29785989 10.4103/ijo.IJO_1274_17 PMC5989503

[6] Nielsen L. S., Skov L. & Jensen H. Visual dysfunctions and ocular disorders in children with developmental delay. I. prevalence, diagnoses and aetiology of visual impairment. Acta Ophthalmologica Scandinavica 85, 149–156 (2007). 17263780 10.1111/j.1600-0420.2006.00867.x

[7] Dutton G. & Bax M. Visual Impairment in Children Due to Damage to the Brain. (John Wiley & Sons, 2010).

[8] Roman-Lantzy C. Cortical Visual Impairment: An Approach to Assessment and Intervention. (American Foundation for the Blind, 2007).

[9] Dutton G. N., McKillop E. C. A. & Saidkasimova S. Visual problems as a result of brain damage in children. British Journal of Ophthalmology 90, 932–933 (2006). 16854832 10.1136/bjo.2006.095349 PMC1857187

[10] Lueck A. H. Cortical or cerebral visual impairment in children: A brief overview. Journal of Visual Impairment & Blindness 104, 585–592 (2010).

[11] Sakki H. E. A., Dale N. J., Sargent J., Perez-Roche T. & Bowman R. Is there consensus in defining childhood cerebral visual impairment? A systematic review of terminology and definitions. British Journal of Ophthalmology 102, 424–432 (2018). 29146757 10.1136/bjophthalmol-2017-310694

[12] Alimović S., Jurić N. & Bošnjak V. M. Functional vision in children with perinatal brain damage. The Journal of Maternal-Fetal & Neonatal Medicine 27, 1491–1494 (2014). 24199646 10.3109/14767058.2013.863863

[13] Boot F. H., Pel J. J. M., van der Steen J. & Evenhuis H. M. Cerebral Visual Impairment: Which perceptive visual dysfunctions can be expected in children with brain damage? A systematic review. Research in Developmental Disabilities 31, 1149–1159 (2010). 20822882 10.1016/j.ridd.2010.08.001

[14] McConnell E. L., Saunders K. J. & Little J.-A. What assessments are currently used to investigate and diagnose cerebral visual impairment (CVI) in children? A systematic review. Ophthalmic and Physiological Optics 41, 224–244 (2021). 33368471 10.1111/opo.12776 PMC8048590

[15] Chandna A., Ghahghaei S., Foster S. & Kumar R. Higher Visual Function Deficits in Children With Cerebral Visual Impairment and Good Visual Acuity. Front. Hum. Neurosci. 15, (2021). 10.3389/fnhum.2021.711873 PMC8636735 34867236

[16] Brosseau-Lachaine O., Gagnon I., Forget R. & Faubert J. Mild traumatic brain injury induces prolonged visual processing deficits in children. Brain Injury 22, 657–668 (2008). 18698516 10.1080/02699050802203353

[17] Mooney S. W. J., Alam N. M. & Prusky G. T. Tracking-Based Interactive Assessment of Saccades, Pursuits, Visual Field, and Contrast Sensitivity in Children With Brain Injury. Front. Hum. Neurosci. 15, (2021). 10.3389/fnhum.2021.737409 PMC8586078 34776907

[18] Chang M., Roman-Lantzy C., O’Neil S. H., Reid M. W. & Borchert M. S. Validity and reliability of CVI Range assessment for Clinical Research (CVI Range-CR): a longitudinal cohort study. BMJ Open Ophthalmology 7, e001144 (2022).

[19] Trovato M. K. Physical Abilities and Mobility Scale: Reliability and Validity in Children Receiving Inpatient Rehabilitation for Acquired Brain Injury. Archives of Physical Medicine and Rehabilitation 94, 1335–1341 (2013). 23254275 10.1016/j.apmr.2012.12.004 PMC3732163

[20] Slomine B. The Cognitive and Linguistic Scale. The Journal of Head Trauma Rehabilitation 21, 419 (2006). 10.1097/01.HTR.0000336841.53338.2f 18815505

[21] Slomine B. S., Grasmick P. H., Suskauer S. J. & Salorio C. F. Psychometric properties of the Cognitive and Linguistic Scale: A follow-up study. Rehabilitation Psychology 61, 328–335 (2016). 27196853 10.1037/rep0000096

[22] Mooney S. W. J., Alam N. M., Hill N. J. & Prusky G. T. Gradiate: A radial sweep approach to measuring detailed contrast sensitivity functions from eye movements. Journal of Vision 20, 17–17 (2020). 10.1167/jov.20.13.17 PMC7774112 33369613

[23] Hill N. J., Mooney S. W. J., Ryklin E. B. & Prusky G. T. Shady: A software engine for real-time visual stimulus manipulation. Journal of Neuroscience Methods 320, 79–86 (2019). 30946876 10.1016/j.jneumeth.2019.03.020 PMC6524778

[24] Hill N. J., Mooney S. W. J. & Prusky G. T. audiomath: A neuroscientist’s sound toolkit. Heliyon 7, e06236 (2021). 33615015 10.1016/j.heliyon.2021.e06236 PMC7881231

[25] Mooney S. W. J. Curveball: A tool for rapid measurement of contrast sensitivity based on smooth eye movements. Journal of Vision 18, 7–7 (2018). 10.1167/18.12.7 PMC6238984 30452585

[26] Harris P. A. Research electronic data capture (REDCap)—A metadata-driven methodology and workflow process for providing translational research informatics support. Journal of Biomedical Informatics 42, 377–381 (2009). 18929686 10.1016/j.jbi.2008.08.010 PMC2700030

[27] DiCesare C. A., Kiefer A. W., Nalepka P. & Myer G. D. Quantification and analysis of saccadic and smooth pursuit eye movements and fixations to detect oculomotor deficits. Behav Res 49, 258–266 (2017). 10.3758/s13428-015-0693-x 26705117

[28] Williams I. M. Cerebral control of saccades and neuropsychological test results after head injury. J Clin Neurosci 4, 186–196 (1997). 18638954 10.1016/s0967-5868(97)90072-2

[29] Lirani-Silva E., Stuart S., Parrington L., Campbell K. & King L. Saccade and Fixation Eye Movements During Walking in People With Mild Traumatic Brain Injury. Front Bioeng Biotechnol 9, 701712 (2021). 34805104 10.3389/fbioe.2021.701712 PMC8602343

[30] Brandt T. & Dieterich M. Pathological eye-head coordination in roll: tonic ocular tilt reaction in mesencephalic and medullary lesions. Brain 110, 649–666 (1987). 3495315 10.1093/brain/110.3.649

[31] Mossman S. & Halmagyi G. M. Partial ocular tilt reaction due to unilateral cerebellar lesion. Neurology 49, 491–493 (1997). 9270583 10.1212/wnl.49.2.491

